# A Nucleotide-Binding Domain Leucine-Rich Repeat Gene Regulates Plant Growth and Defense Against Chewing Herbivores

**DOI:** 10.3390/plants13233275

**Published:** 2024-11-22

**Authors:** Chen Qiu, Xiaochen Jin, Yumiao Zhao, Peng Kuai, Yonggen Lou

**Affiliations:** 1 State Key Laboratory of Rice Biology & Ministry of Agriculture Key Laboratory of Agricultural Entomology, Key Laboratory of Biology of Crop Pathogens and Insects of Zhejiang Province, Institute of Insect Sciences, Zhejiang University, Hangzhou 310058, China; cqiu2019@163.com (C.Q.); 12116098@zju.edu.cn (X.J.); zhaoyumiaoi@163.com (Y.Z.); 2 Hainan Institute, Zhejiang University, Sanya 572025, China

**Keywords:** rice, *OsPik-2-like*, *Cnaphalocrocis medinalis*, jasmonic acid, jasmonoyl–isoleucine, trypsin protease inhibitors

## Abstract

Plant nucleotide-binding leucine-rich repeat immune receptor genes (NLRs) play an important role in plant defenses against pathogens, pathogenic nematodes, and piercing–sucking herbivores. However, little is known about their functions in plant defenses against chewing herbivores. Here, we identified a plasma membrane-localized coiled-coil-type NLR protein, OsPik-2-like, whose transcript levels were induced by the infestation of rice leaf folder (LF, *Cnaphalocrocis medinalis*) larvae, and by treatment with mechanical wounding. Knocking out *OsPik-2-like* in rice increased the LF-induced levels of jasmonic acid (JA) and jasmonoyl–isoleucine (JA-Ile), the activity of trypsin protease inhibitors (TrypPIs), and the basal levels of some flavonoids, which in turn decreased the performance of LF larvae. Moreover, knocking out *OsPik-2-like* reduced plant growth. These findings demonstrate that OsPik-2-like regulates the symbiosis between rice and LF by balancing plant growth and defense.

## 1. Introduction

Plants are constantly being challenged by a variety of stresses, including herbivores. As a consequence, plants have evolved complicated strategies to cope with herbivore infestation. When attacked by herbivores, plants employ two tiers of immune receptors—plasma membrane-localized pattern recognition receptors (PRRs) and intracellular nucleotide-binding domain leucine-rich repeat receptors (NLRs)—to initiate specific defense responses [1,2,3]. PRRs mainly recognize plant-derived damage-associated molecular patterns (DAMPs) or herbivore-associated molecular patterns (HAMPs, also called herbivore-associated elicitors) to activate pattern-triggered immunity (PTI) [2,4], whereas NLRs mainly perceive herbivore-secreted effectors to initiate effector-triggered immunity (ETI) [5,6]. After recognizing patterns/effectors, plants activate a series of defense responses, including calcium influx, the production of reactive oxygen species (ROS), the activation of mitogen-activated protein kinases (MAPKs), and signaling pathways mediated by phytohormones (such as jasmonic acid (JA), salicylic acid (SA), ethylene (ET), and abscisic acid (ABA)), and the accumulation of defense compounds. Acting together, these responses enhance the direct and indirect resistance of plants to herbivores [2,7].

Plant NLRs are usually composed of three conserved domains: a variable N-terminal domain; a central NB-ARC (nucleotide-binding adaptor shared by Apaf1, certain resistance genes, and a CED4) domain; and a C-terminal LRR (leucine-rich repeat) domain [8]. Based on their N-terminal domains, plant NLRs are divided into three classes, including Toll/interleukin-1 receptor/R protein (TIR)-type NLRs (TNLs); coiled-coil (CC)-type NLRs (CNLs); and resistance to powdery mildew 8 (RPW8)-type NLRs (RNLs) [8]. Numerous studies have revealed that NLRs usually regulate plant disease resistance by forming homomultimers or heteromultimers [8,9]. For example, *PigmR* (*Pigm Resistant*) and *PigmS* (*Pigm Susceptible*) are functional gene pairs identified at the rice *Pigm* locus for rice blast resistance. PigmR forms homologous dimers conferring broad-spectrum disease resistance to rice, but severely decreasing rice yields in the process; however, PigmS binds to PigmR to form heterodimers, thereby inhibiting PigmR-mediated broad-spectrum disease resistance and offsetting the negative effect of PigmR on rice yields [10]. In *Arabidopsis* and tobacco plants, ZAR1 (HopZ-activated resistance 1) interacts with multiple members of the receptor-like cytoplasmic kinase (RLCK) subfamily to form immune receptor complexes; these sense the pathogen-derived effectors and trigger immune responses [11,12,13,14]. For instance, the ZAR1 resistosome in *Arabidopsis*—a heterodimer composed of ZAR1, the resistance-related kinase 1 (RKS1), and the PBL2^UMP^ (the acetylated form of PBL2 by the effector protein AvrAC of *Xanthomonas campestris* pv. *campestris*)—directly enters the lipid bilayer of the plasma membrane of cells, where it acts as a Ca^2+^ channel and promotes Ca^2+^ influx, thereby triggering ROS production and cell death [14].

Recently, NLRs have also been reported to play an important role in plant herbivore resistance [3]. For example, several NLR genes involved in plant resistance to aphids have been identified and cloned, such as *Vat* in *Cucumis melo*, *Ra* in *Lactuca sativa*, and *AIN* (*Acyrthosiphon-induced necrosis*) in *Medicago truncatula* [15,16,17,18]. Also, the resistance gene *Mi1.2* in *Lycopersicon peruvianum* confers broad-spectrum resistance to root-knot nematodes, whitefly (*Bemisia tabaci*), and psyllid (*Bactericerca cockerelli*) on plants [19,20]. When *Pieris brassicae* egg deposition-induced hypersensitive response (HR)-like cell death was genetically mapped in the black mustard *Brassica nigra*, a cluster of TNL genes was identified as a potential source of R genes; these genes may regulate the ability of plants to kill herbivore eggs [21]. In rice, two CNL genes, *Brown planthopper resistance 9* (*Bph9*) and *Bph14*, are well known to confer BPH resistance on rice: *Bph9* regulates rice BPH resistance by affecting JA, SA, and ET-mediated signaling pathways, and *Bph14* positively mediates the resistance of rice to BPH by promoting SA and ROS-mediated defenses [22,23,24,25]. Further studies proved that the CC and NB domains, as well as the full length of Bph14, interact with two WRKY proteins (OsWRKY46 and OsWRKY72); this interaction stabilizes the two WRKY proteins and enhances their transactivation activity, thereby increasing the expression of callose-biosynthesis genes and the deposition of calloses in the phloem of rice leaf sheaths [26]. Moreover, Bph14 interacts with the BPH-derived effector BISP (Bph14-interacting salivary protein) to activate ETI, which markedly increased rice resistance to BPH [27]. However, our understanding of the role of NLRs in plant defense against herbivores and their underlying mechanisms is limited.

Rice (*Oryza sativa* L.), one of the primary food crops in the world, is often seriously damaged in the field by multiple herbivores, including the rice leaf folder (LF, *Cnaphalocrocis medinalis*) [28]. LF larvae fold rice leaves longitudinally and feed on the green mesophyll tissues of these folded leaves, actions that reduce both the photosynthetic productivity and grain yield of rice plants [29]. It has been reported that the infestation of LF larvae activates defense-related signaling pathways mediated by JA, SA, ET, and H_2_O_2_ in rice; these signaling pathways jointly modulate the expression of defense genes and the accumulation of defense compounds, such as trypsin protease inhibitors (TrypPIs) and phenolamines [30,31]. Although a few NLRs have been reported to be involved in the resistance of rice to piercing–sucking herbivores, as stated above, whether NLRs participate in rice defense against chewing herbivores, such as LF, remains largely unknown.

In this study, we isolated a rice CNL gene *OsPik-2-like* (XM_015756755.2), which was induced by LF larval infestation [31], and investigated its function in interactions between rice and LF. By combining molecular tools, chemical analysis, and bioassays, we found that *OsPik-2-like* negatively regulates the biosynthesis of LF-induced JA and JA-Ile, the activity of induced TrypPIs, and the resistance of rice to LF, suggesting that NLRs participate in rice–LF interactions.

## 2. Results

### 2.1. Characterization of OsPik-2-like

**Figure 1:**
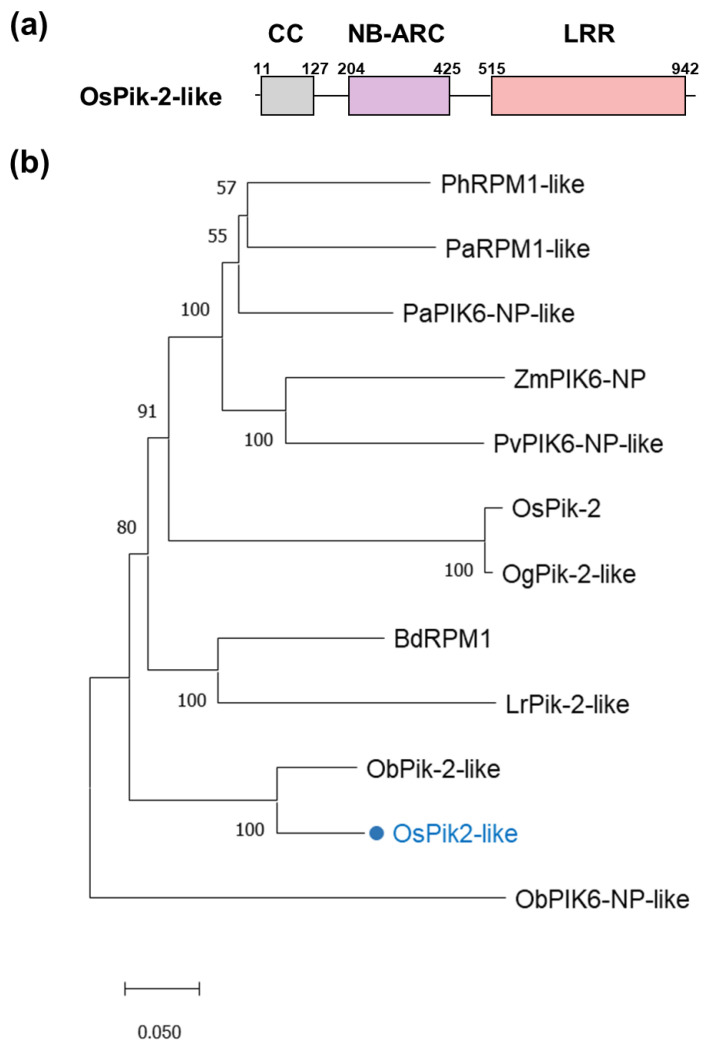
Structure and phylogenetic relationship of OsPik-2-like. (**a**) Schematic structure of OsPik-2-like. (**b**) Sequence alignment of OsPik-2-like and its homologs. Species abbreviations are included before the protein names: Bd, *Brachypodium distachyon*; Lr, *Lolium rigidum*; Ob, *Oryza brachyantha*; Og, *Oryza glaberrima*; Os, *Oryza sative*; Pa, *Phragmites australis*; Ph, *Panicum hallii*; Pv, *Panicum virgatum*; and Zm, *Zea mays*. Plant species and accession numbers from the NCBI database are as follows: BdRPM1, XP_003576018.1; LrPik-2-like, XP_047085044.1; ObPIK6-NP-like, XP_040383595.1; ObPik-2-like, XP_006660848.1; OgPik-2-like, XP_052139461.1; OsPik-2, XP_015619167.2; OsPik-2-like, XP_015612241.1; PaPIK6-NP-like, XP_062198511.1; PaRPM1-like, XP_062193254.1; PhRPM1-like, XP_025803740.1; PvPIK6-NP-like, XP_039795950.1; ZmPIK6-NP, XP_008652943.2. The blue dot indicates OsPik-2-like. The scale bar represents 0.05 amino acid substitutions per site in the primary structure.

Using transcriptome data [31], we determined that a putative CNL gene was up-regulated by herbivore infestation. The full-length cDNA sequence obtained from a cDNA library of rice variety XS11 using the reverse-transcription polymerase chain reaction (RT-PCR) was identical to the sequence of a rice disease resistance gene *OsPik-2-like* (annotated in the Genbank database, https://www.ncbi.nlm.nih.gov/genbank/, under the ID: XM_015756755.2; accessed 30 August 2023). The gene includes an open-reading frame of 2,892 bp that encodes a protein of 963 amino acids with a predicted molecular weight of 109.03 kDa (Figure S1). The protein harbored three conserved domains: a CC domain, a NB-ARC domain, and a LRR domain (Figure 1a). OsPik-2-like shared high similarity with CNL proteins in other plants, including ObPik-2-like (88.30% identity) in *Oryza brachyantha*, BdRPM1 (72.28% identity) in *Brachypodium distachyon,* and PaPIK6-NP-like (71.06% identity) in *Phragmites australis* (Figure 1b). These results showed that OsPik-2-like belongs to the CNL protein family.

**Figure 2:**
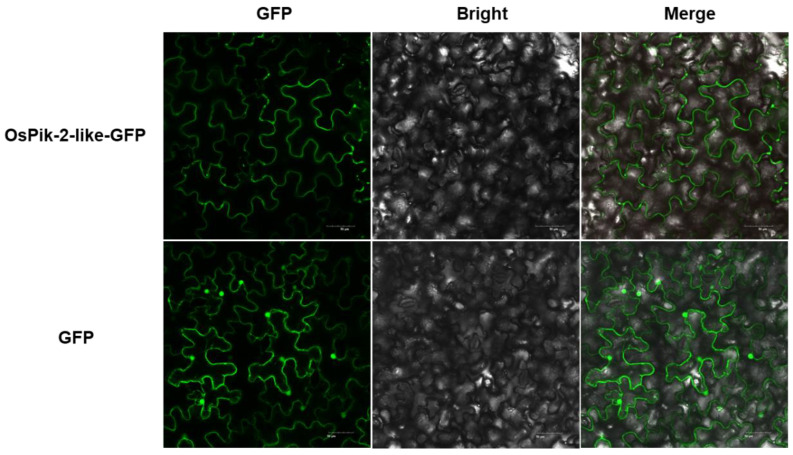
Subcellular localization of OsPik-2-like. Subcellular localization of the fused OsPik-2-like-GFP or GFP in *N. benthamiana* leaf cells. OsPik-2-like-GFP, green fluorescent protein (GFP) fluorescence from OsPik-2-like-GFP; Bright, bright field; Merged, the merged image of OsPik-2-like-GFP or GFP and Bright. Bar = 50 μm.

To elucidate the subcellular localization of OsPik-2-like, we generated the construct *35S::OsPik-2-like-EGFP* (the enhanced green fluorescent protein was fused to OsPik-2-like), which was driven by a *CaMV 35S* promoter. Then, the construct was transiently expressed in *Nicotiana benthamiana* leaves using *Agrobacterium tumefaciens*-mediated transformation. As shown in Figure 2, compared with the wide distribution of the control EGFP, the OsPik-2-like-EGFP mainly localized to plasma membranes of tobacco leaf cells, indicating that OsPik-2-like was a plasma membrane (PM)-localized CNL protein.

**Figure 3:**
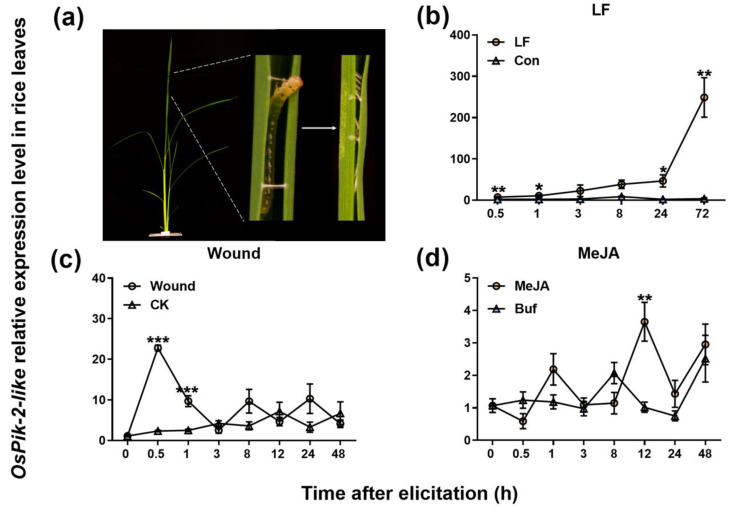
Relative expression levels of *OsPik-2-like* in rice leaves after different treatments. (**a**) Phenotypes of rice plants that had been infested by one 3rd-instar LF larva. (**b**–**d**) Mean transcript levels (±SE, n = 3~6) of *OsPik-2-like* in rice leaves that were infested with one 3rd LF larva (LF) (**b**), or punctured by a fabric pattern wheel rolling (Wound) (**c**), or treated with methyl jasmonate (MeJA) by root absorption (**d**). Con, non-treated plants; Buf: plants treated with the same concentration of ethanol in the nutrient solution as MeJA treatment. Asterisks represent significant differences between the treatments and controls at each time point (* *p* < 0.05, ** *p* < 0.01, and *** *p* < 0.001, Student’s *t*-tests or *t* tests with Welch’s correction).

Quantitative real-time PCR (qRT-PCR) revealed that the constitutive transcript levels of *OsPik-2-like* in rice leaves were low. However, when rice plants (leaf blades) were infested by LF larvae (Figure 3a,b), the levels of *OsPik-2-like* transcripts in leaf blades were rapidly (starting 0.5 h after infestation) and lastingly up-regulated; the levels, which peaked at 72 h, were about 70-fold higher than the levels in control plants (Figure 3b; Table S1). Mechanical wounding also quickly (0.5 h after treatment) induced the expression of *OsPik-2-like* (levels, which peaked at 0.5 h, were about 10-fold higher than levels in control plants); however, the levels of *OsPik-2-like* transcripts rapidly declined to those of controls 3 h after treatment (Figure 3c; Table S1). MeJA treatment only slightly induced the expression of *OsPik-2-like* at 12 h after treatment (Figure 3d; Table S1). These results suggest that *OsPik-2-like* might be involved in LF-induced rice defense responses.

### 2.2. Knocking out OsPik-2-like in Rice

**Figure 4:**
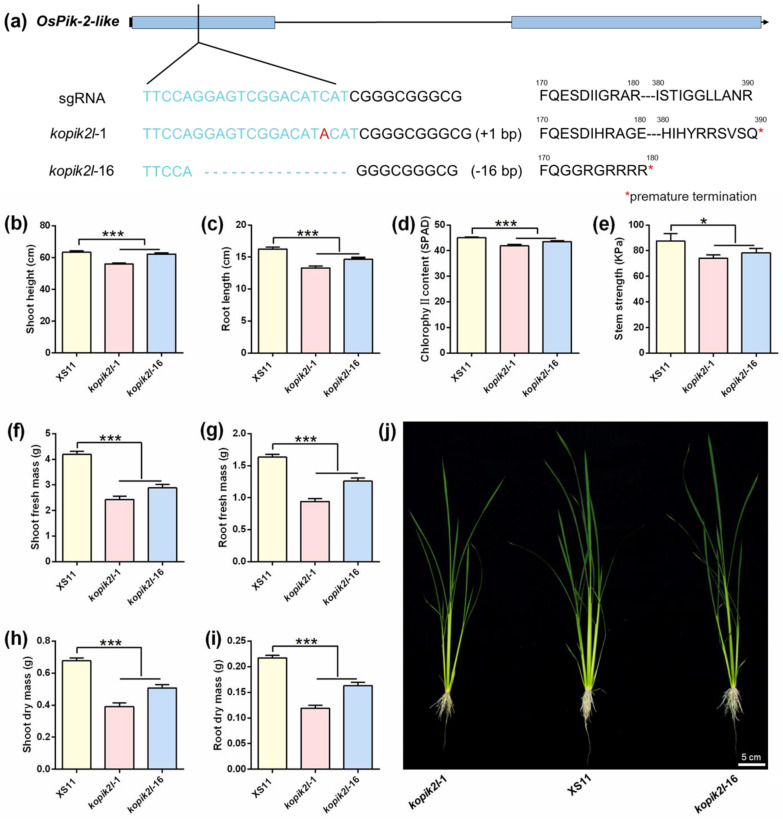
*OsPik-2-like* gene-knockout rice lines and their growth phenotypes. (**a**) Sanger sequencing analysis of mutation patterns in the target site of *OsPik-2-like* gene in the wild-type (WT) and *OsPik-2-like* knockout rice lines. Blue boxes represent exons. The sgRNA sequence that specifically targets *OsPik-2-like* is indicated. The mutations, an ‘‘A’’ insertion in line *kopik2l-*1 and a 16 bp deletion in *kopik2l-*16, lead to premature translational termination of OsPik-2-like. (**b**–**i**) Mean shoot height (**b**), root length (**c**), chlorophyll content (**d**), stem strength (**e**), shoot fresh weight (**f**), root fresh weight (**g**), shoot dry weight (**h**), and root dry weight (**i**) (+SE, n = 20) of WT plants and ko-*pik2l* lines at 30 days old in the phytotron. Asterisks indicate significant differences in ko-*pik2l* lines compared with WT plants evaluated by Bayesian analysis of variance (* *p* < 0.05 and *** *p* < 0.001). (**j**) The phenotype of 30-day-old WT and ko-*pik2l* lines in the phytotron.

To explore the function of *OsPik-2-like* in interactions between rice and LF, we obtained two T-DNA-free homozygous rice lines with knocked-out *OsPik-2-like* in the variety XS11 using a CRISPR/Cas9-mediated gene-editing system (ko-*pik2l*: *kopik2l-*1 and *kopik2l-*16). As shown in Figure 4a, compared with the target sequence of *OsPik-2-like* gene in WT plants, the line *kopik2l-*1 displayed a single “A” insertion and the line *kopik2l-*16 showed a 16-bp deletion (Figure 4a). DNA sequencing analysis revealed no mutations of off-target sites (such mutations had been predicted by the CRISPR-P server, http://cbi.hzau.edu.cn/crispr/, accessed 25 June 2023), in ko-*pik2l* lines (Figure S2), suggesting that the CRISPR/Cas9-based *OsPik-2-like*-knockout was specific.

We investigated the effect of knocking out *OsPik-2-like* on plant growth. Knocking out *OsPik-2-like* reduced shoot height (by 11.75% in *kopik2l-*1 and 1.84% in *kopik2l-*16, compared with WT plants), root length (by 18.18% and 9.91%), chlorophyll content in leaves (by 7.15% and 3.47%), stem strength (by 15.34% and 10.7%), shoot fresh mass (by 42.24% and 31.21%) and shoot dry mass (by 42.17% and 25.14%), and root fresh mass (by 42.47% and 22.99%) and root dry mass (by 45.28% and 25.01%) of 30-day-old plants (Figure 4b–i; Table S2). These data suggest that *OsPik-2-like* positively regulated rice growth.

### 2.3. Knocking out OsPik-2-like-Enhances LF-Induced JA and JA-Ile Levels

**Figure 5:**
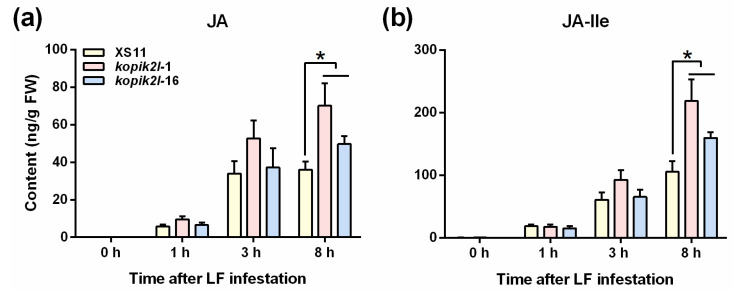
Knocking out *OsPik-2-like* enhanced LF-induced JAs levels in rice leaves. Mean levels (+SE, n = 4~6) of JA (**a**) and JA-Ile (**b**) in WT plants and ko-*pik2l* lines that were individually infested by a 3rd-instar LF larva on the first fully expanded leaf at the indicated time points. Asterisks indicate significant differences in ko-*pik2l* lines compared with WT plants evaluated by Bayesian analysis of variance (* *p* < 0.05).

The JA-mediated signaling pathway plays a central role in rice defenses against chewing herbivores [32]. Hence, we determined the levels of JA and JA-Ile in WT plants, as well as ko-*pik2l* lines before and after LF infestation. Consistent with previous studies [31,33], LF infestation significantly increased the contents of JA and JA-Ile in rice leaf blades in WT plants (Figure 5; Table S3). Knocking out *OsPik-2-like* did not influence the constitutive levels of JA and JA-Ile in rice leaves. However, it did enhance the induced levels of JA and JA-Ile, especially JA-Ile, in rice leaves 8 h after LF infestation: the LF-induced JA-Ile levels in the two ko-*pik2l* lines *kopik2l-*1 and *kopik2l-*16 were about 2- and 1.5-fold higher than those in WT plants (Figure 5; Table S3).

We also found that LF larval infestation (at 8 h) enhanced the content of 3-indoleacetic acid (IAA) in rice leaf blades. However, no difference was observed in IAA content between WT and ko-*pik2l* lines (Figure S3a; Tables S3 and S4). These results indicate that the JA signaling pathway might be involved in *OsPik2-like*-mediated rice defenses against LF.

### 2.4. Knocking out OsPik-2-like Enhances the Level of Defense Compounds and Rice Resistance to LF

**Figure 6:**
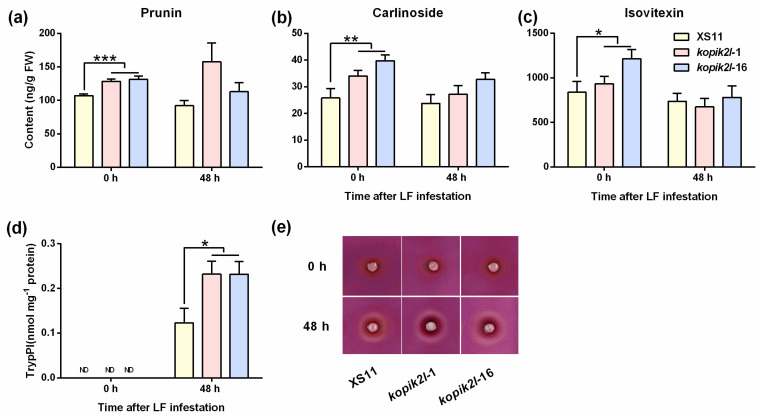
Flavonoid levels and TrypPI activity in the leaves of WT plants and ko-*pik2l* lines. Mean concentrations (+SE, n = 5~6) of prunin (**a**), carlinoside (**b**), isovitexin (**c**), and mean TrypPI activity (+SE, n = 7) (**d**) in WT plants and ko-*pik2l* lines that were individually infested by a 3rd-instar LF larva on the first fully expanded leaf for 0 h or 48 h. Asterisks indicate significant differences in ko-*pik2l* lines compared with WT plants evaluated by Bayesian analysis of variance (* *p* < 0.05, ** *p* < 0.01 and *** *p* < 0.001). (**e**) Representative pictures of TrypPI after different treatments measured by radial diffusion assay.

Plants employ multiple defense compounds to limit the population development of herbivores, such as flavonoids, phenolamides, and TrypPIs [32]. To evaluate whether *OsPik-2-like* regulates the production of defense compounds in rice plants, we examined the content of flavonoids and TrypPIs in WT plants and in ko-*pik2l* lines before and after LF infestation. Knocking out *OsPik-2-like* in rice constitutively increased the contents of three flavonoids—prunin, carlinoside, and isovitexin (Figure 6a–c; Table S5)—but decreased the contents of two flavonoids—astragalin and luteolin 7-*O*-glucoside (Figure S3b,c; Table S5). After LF larval infestation, knocking out *OsPik-2-like* also decreased the contents of five flavonoids: astragalin (by 49.83% in *kopik2l*-1 and 59.17% in *kopik2l*-16 compared to WT plants), luteolin 7-*O*-glucoside (by 49.89% and 59.76%), luteolin (by 65.71% and 72.89%), sakuranetin (by 100% and 100%), and isoquercitrin (by 32.61% and 28.04%) (Figure S3b–f; Table S5). Consistent with the results reported previously [31], the basal level of TrypPIs in rice leaves of WT plants was extremely low. However, when plants were infested by LF larvae, the TrypPI activity in leaves was markedly enhanced (Figure 6d,e; Table S5). Knocking out *OsPik-2-like* did not influence the basal activity of TrypPIs in plant leaf blades, but it did significantly enhance the LF-induced activities of TrypPIs in leaf blades (Figure 6d,e; Table S5).

**Figure 7:**
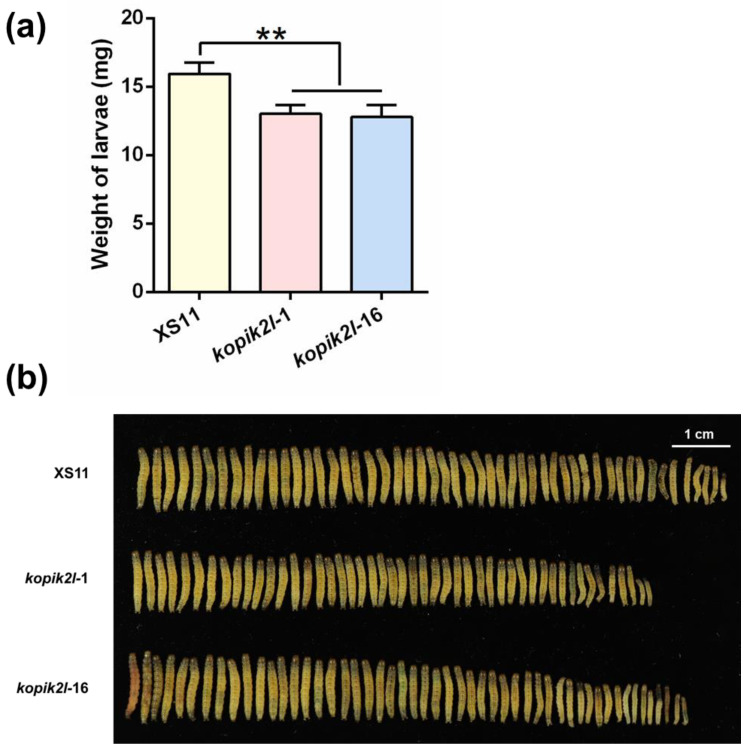
*OsPik-2-like* negatively regulates the resistance of rice to LF. (**a**) Mean larval mass (+SE, n = 48~51) of LF fed on WT plants and ko-*pik2l* lines for 11 days. (**b**) Phenotypes of LF larvae fed on different lines on day 11. Asterisks indicate significant differences in ko-*pik2l* lines compared with WT plants evaluated by Bayesian analysis of variance (** *p* < 0.01).

Given that knocking out *OsPik-2-like* in rice influenced the JA signaling pathway and the levels of defense compounds in plants, we asked whether knocking out *OsPik-2-like* affected rice defense against LF. As expected, LF larvae fed on ko-*pik2l* plants gained significantly less mass compared with those fed on WT plants: by day 11, the mass of LF larvae fed on the *kopik2l-*1 and *kopik2l-*16 plants decreased by about 18% and 20%, respectively, compared with the mass of larvae fed on WT plants (Figure 7; Table S6). These results indicate that *OsPik-2-like* negatively regulates rice defense against LF larvae.

## 3. Discussion

Plant NLRs have widely been reported to act as resistance proteins that sense pathogen/herbivore-derived effectors, thereby triggering ETI. However, their role in plant defense responses to chewing herbivores remains poorly understood [15,16,17,18,19,20,21,22,23,24,25]. In this study, we revealed that a CNL protein, OsPik-2-like, plays an important role in LF larvae-induced rice defenses. First, OsPik-2-like localized to PM, and its transcript levels were significantly induced by LF larval infestation and mechanical wounding. Second, knocking out *OsPik-2-like* decreased the shoot mass, root mass leaf chlorophyll content, and stem strength of plants. Third, knocking out *OsPik-2-like* enhanced the LF-induced levels of JA and JA-Ile, as well as the activity of TrypPIs in rice, which in turn reduced the growth of LF larvae. Our findings demonstrate that *OsPik-2-like* positively regulates plant growth, but negatively mediates the resistance of rice plants to LF.

NLRs play an important role in regulating defense-related signaling pathways [3]. Two NLRs, *Sw-5b* and *Sl5R-1*, from *Solanaceae*, for instance, have been reported to activate the JA signaling pathway and then increase plant resistance to infection by *tomato spotted wilt orthotospovirus* (TSWV) [34,35]. In rice, *Bph9* and *Bph14*, two NLRs, have been reported to negatively regulate the expression of genes related to JA biosynthesis, such as genes encoding allene oxide synthase 2 (*OsAOS2*) and lipoxygenase (*OsLOX*); moreover, the levels of JA and JA-Ile in plants carrying *Bph9* or *Bph14* were significantly lower than those in WT plants in response to BPH infestation [22,25]. Here, we observed that knocking out *OsPik-2-like* significantly increased the LF-elicited levels of JA and JA-Ile in rice leaf blades (Figure 5). Moreover, LF larval infestation up-regulated the transcript levels of *OsPik-2-like*, especially late in the process. These data suggest that, like *Bph9* and *Bph14*, *OsPik-2-like* negatively modulated the biosynthesis of LF-induced JA and JA-Ile in rice. These results indicate that LF was able to suppress the JA-mediated rice defense by inducing the expression of *OsPik-2-like*. Further studies should elucidate the mechanism underlying the *OsPik-2-like*-mediated regulation of JA biosynthesis. It would also be interesting to know whether and how other defense-related signaling pathways are modulated by OsPik-2-like.

Why rice plants up-regulate the expression of *OsPik-2-like* at late stages of infestation by LF larvae, given that *OsPik-2-like* negatively regulated rice defense, remains a question. It may be that rice plants are trying to avoid the autotoxicity caused by excessive levels of defenses [36,37] and to restore growth. Given that *OsPik-2-like* suppressed the biosynthesis of LF-induced JA and JA-Ile, and that knocking out *OsPik-2-like* decreased plant growth (Figure 4 and Figure 5), the up-regulation of *OsPik-2-like* at the late stage of LF larval infestation caused plants to maintain appropriate levels of defenses. Moreover, it made plants regrow quickly. In rice, several such genes, such as *OsMPK20-5* [38] and *OsLRR2* [39], have been reported. Alternatively, it may be related to herbivore decoy strategies [40]. Thus far, many CNLs have been reported to regulate plant disease resistance [3]. In rice, two CNLs, *Pik-1* and *Pik-2*, have been reported to enhance rice resistance to *Magnaporthe oryzae* (syn. *Pyricularia oryzae*) expressing the *AvrPik* effector [41,42,43]. Because many symbiotic microbe species live in herbivores [44], it may be that LF larvae regulate the expression of *OsPik-2-like* by secreting one of these microbes during their feeding, thereby suppressing the JA-mediated defense in rice. Further research should clarify this mechanism. Moreover, how OsPik-2-like influences rice growth, including which plant growth-related signaling pathways are regulated by OsPik-2-like, should also be elucidated.

The JA signaling pathway plays a central role in regulating the biosynthesis of multiple defense compounds [31,33]. For example, silencing or knocking out genes related to JA biosynthesis (such as herbivore-induced rice type 2 13-LOX gene, *OsHI-LOX*, and the allene oxide cyclase gene, *OsAOC*) or signaling (JA receptor gene, rice *CORONATINE-INSENSITIVE1* (*OsCOI1*) and the core JA-responsive transcriptional factor *OsMYC2*) in rice reduces plant resistance to LF by impairing the activity of TrypPIs and the accumulation of some phenolamines in rice plants [31,33]. Therefore, the observed results—namely, that knocking out *OsPik-2-like* enhanced the activity of LF-induced TrypPIs in plants—were related, at least in part, to the increase in the levels of LF-induced JA and JA-Ile in plants. Interestingly, we observed that knocking out *OsPik-2-like* increased the basal levels of three flavonoids and decreased the basal levels of two flavonoids; moreover, it also decreased LF-induced levels of five flavonoids (Figure 6). Although some researchers have reported that the JA signaling pathway positively modulates the production of herbivore-induced flavonoids [45], these results indicate that the regulation of flavonoid biosynthesis was complex and that other signaling pathways were also involved in this process. It has been reported that signaling pathways mediated by brassinosteroids, SA, and ET also regulate the biosynthesis of flavonoids in plants [46,47,48]. Future research should elucidate which other signaling pathways regulated by OsPik-2-like mediate the biosynthesis of these compounds in rice.

TrypPIs and some flavonoids are important defense compounds against herbivores. TrypPIs, for example, have been reported to suppress the growth and development of chewing herbivores, including LF, by inhibiting the activity of digestive enzymes in their midgut [30,31,49]. Some flavonoids (including prunin, carlinoside, schaftoside, and its isomers, isoschaftoside and neoschaftoside) impede the feeding, survival, and development of rice planthoppers [50,51,52], although no flavonoids have been reported to affect the performance of LF larvae. Moreover, two isovitexin-derived compounds, isovitexin 2″-O-(6‴-(*E*)-feruloyl) glucoside and isovitexin-2′-*O*-β-[6-O-E-*p*-coumaroylglucopyranoside], decrease the probing responses of *Nephotettix cincticepts* [53] and the fecundity of *Helicoverpa armigera*, respectively [54]. Hence, the enhanced resistance of ko-*pik2l* plants to LF larvae is probably related to the increase in the levels of defense compounds, such as TrypPIs and some flavonoids. Future research should clarify which compounds are responsible for the performance of LF larvae on ko-*pik2l* plants.

In summary, our results demonstrate that the PM-localized rice CNL protein, OsPik-2-like, plays an important role in regulating the interaction between rice and LF larvae. When infested by LF larvae, plants perceive signals derived from the herbivore and then initiate defense-related signaling pathways, such as the JA signaling pathway. These changes enhance the expression of defense-related genes and the level of defense compounds, such as TrypPIs, in turn decreasing the performance of LF larvae. The up-regulation of *OsPik-2-like* in rice at late stages of LF larval infestation not only reduces plant defense by suppressing the JA signaling pathway, but also promotes plant regrowth. Both consequences may be beneficial for both plants and herbivores. Plants may avoid the autotoxicity associated with excessive defenses and also experience regrowth; herbivores may improve growth due to decreased plant defense. Our study provides an interesting example of how a single gene can act as a modulator for the symbiotic relationship between plants and herbivores, balancing plant growth and defense. From an application point of view, this study shows how specific genes can be edited in plants to control insect pests.

## 4. Materials and Methods

### 4.1. Plants and Insects

The *japonica* rice variety XiuShui 11 (XS11) was used as the wild type (WT) in this study. The gene-knockout rice lines of *OsPik-2-like* (ko-*pik2l*: *kopik2l-*1 and *kopik2l-*16) were generated and screened as below. Seeds of WT and ko-*pik2l* lines were pre-germinated in plastic culture dishes (diameter 90 mm, height 15 mm) in a climate incubator under the following conditions: 28 ± 2 °C, 60% relative humidity, and 14/10 h light/dark cycle. Ten days later, rice seedlings were transplanted to 25 L hydroponic boxes (length 51 cm, width 35 cm, and height 17 cm) brimming with rice nutrient solution [55] and cultivated in a phytotron (26 ± 2 °C, 14 h light phase, 60% relative humidity). Twenty-five-day-old rice plants were individually transferred into plastic pots (diameter 7 cm, height 9.5 cm) filled with 350 mL of nutrient solution. Plants were used for experiments 4 d after transplantation.

Colonies of LF were originally obtained from a rice paddy in Hangzhou, China, and subsequently reared with wheat seedlings in a climate chamber (26 ± 2 °C, 14 h light phase, and 65 ± 10% relative humidity) for more than 30 generations.

### 4.2. Plant Treatment

For LF treatments, a third-instar LF larva was starved for 2 h, then placed on the youngest fully expanded leaf of a plant. Untreated plants were used as controls. For mechanical wounding in rice leaves, the youngest fully expanded leaf was punctured by rolling a fabric pattern wheel over it [45]. Untreated plants were used as controls. For mechanical wounding in rice leaf sheaths, aerial parts 2 cm high from the roots of rice plants were individually punctured 200 times using an insect needle (length 40 mm and diameter 0.45 mm; Yuxiu, Taizhou, China). Untreated plants were used as controls. For treatment with methyl jasmonate (MeJA) (Sigma-Aldrich, Darmstadt, Germany), MeJA was dissolved in 1 mL of absolute ethyl alcohol and then added to the nutrient solution to make its concentration 100 μM, as stated in previous studies [56]. Plants cultivated in the nutrient solution with an equal volume of solvent were used as controls.

### 4.3. RNA Extraction and Quantitative Real-Time PCR (qRT-PCR)

Total RNA was extracted from rice samples using FastPure^®^ Universal Plant Total RNA Isolation Kit (Vazyme, Nanjing, China) following the manufacturer’s instructions. One microgram of total RNA was then reverse-transcribed by using HiScript^®^ II Q RT SuperMix for qPCR (+gDNA wiper) (Vazyme, Nanjing, China) according to the manufacturer’s instructions. QRT-PCR assays were performed with Taq Pro Universal SYBR qPCR Master Mix (Vazyme, Nanjing, China) on the CFX96 Real-Time PCR System (Bio-Rad, Richmond, CA, USA). With the *OsACTIN* (TIGR ID: LOC_Os03g50885) as an internal control, the relative expression levels of tested genes were analyzed by the −2^ΔΔCt^ method [56]. All primers used in the qRT-PCR assays are listed in Table S7.

### 4.4. Isolating OsPik-2-like

Four micrograms of total RNA extracted from WT plants was reverse-transcribed to first-strand cDNAs using PrimeScript™ IV cDNA Synthesis Mix (TaKaRa, Dalian, China). Then, the opening read frame of *OsPik-2-like* (XM_015756755.2) was PCR-amplified from the cDNA library using specific primers OsPik-2-like-CDS-F (5′-TCTCCGCCATTTACACCCAC-3′) and OsPik-2-like-CDS-R (5′-TGCCAAAGAGGAACATCGGA-3′), and the KOD FX polymerase (TOYOBO, Shanghai, China) following the manufacturer’s instructions. PCR products were subsequently cloned into the pEASY^®^-Blunt Zero cloning vector (TransGen, Beijing, China) and sequenced. The primers used in this study are listed in Table S8.

### 4.5. Structure and Phylogenetic Analysis of OsPik-2-like

The conserved domains of the deduced OsPik-2-like protein were analyzed using CD-search (https://www.ncbi.nlm.nih.gov/Structure/cdd/wrpsb.cgi; accessed on 30 August 2023). The homologs of OsPik-2-like from different plant species were identified using the BLASTP plug-in in the NCBI online database (https://blast.ncbi.nlm.nih.gov/Blast.cgi; accessed on 24 June 2024). The amino acid sequences of OsPik-2-like and their homologs from other plant species were downloaded from the NCBI website. Then, multiple sequence alignment was carried out using ClustalW program in Molecular Evolutionary Genetics Analysis (MEGA, version 11.0.13) as described previously [56]. A neighbor-joining phylogenetic tree was constructed by MEGA with 1000 bootstrap replicates using the default parameters as described [56].

### 4.6. Subcellular Localization Assay

For subcellular localization, the full-length coding sequence of *OsPik-2-like* without the termination codon was inserted into the pCAMBIA1301-EGFP vector [39] using ClonExpress^®^ II One Step Cloning Kit C112 (Vazyme, Nanjing, China), yielding a *35S::OsPik-2-like-EGFP* construct. The constructed plasmid was then transformed into *A. tumefaciens* strain EHA105 by electroporation. *A. tumefaciens*—cultivated overnight and carrying the OsPik-2-like-EGFP construct—was injected into leaves of 4-week-old *N. benthamiana* plants as previously described [57]. Two days after infiltration, the EGFP fluorescence images were observed and recorded by Olympus FV3000 confocal laser scanning microscope (Olympus, Tokyo, Japan) at an excitation wavelength of 488 nm and an emission wavelength of 507 nm. The primers used in this study are listed in Table S8.

### 4.7. Generating and Characterizing OsPik-2-like Gene-Knockout Rice Plants

To generate *OsPik-2-like* gene-knockout rice lines, the target sequence (5′-TTCCAGGAGTCGGACATCAT-3′) was introduced into pLYsgRNAOsU6b to yield rice U6b promoter-driven single-guide RNA (sgRNA). The sgRNA expression cassette was then introduced into the plant CRISPR-Cas9 binary vector pYLCRISPR/Cas9Pubi-H [58]. The T-DNA was inserted into XS11 using *A. tumefaciens* (EHA105)-mediated transformation. Plants with mutations but lacking the T-DNA were screened by target DNA sequencing and hygromycin resistance gene identification using the method described in [39]. Two homozygous *OsPik-2-like* gene-knockout (ko-*pik2l*) lines, *kopik2l-*1 and *kopik2l-*16, were used for all experiments. Potential off-target sites were predicted by CRISPR-GE (http://skl.scau.edu.cn/home/, accessed 13 August 2023) and identified by target DNA sequencing using the specific primers listed in Table S9.

### 4.8. Measuring Plant Growth Parameters

Thirty-day-old WT plants and *OsPik-2-like* gene-knockout lines were used to measure plant growth parameters, which included root length, shoot height, root fresh mass, root dry mass, shoot fresh mass, shoot dry mass, stem strength, and chlorophyll content. To determine stem strength, the aerial parts (2 cm high from plant root) of rice plants were measured with a plant-stem-strength meter [59], YYD-1 (Top Cloud-Agri, Hangzhou, Zhehjiang, China). To determine chlorophyll content, the middle location of the youngest fully expanded leaves from each plant was measured with a chlorophyll meter [56], SPAD-502 Plus (Konica Minolta, Tokyo, Japan). Each experiment was replicated 20 times.

### 4.9. Herbivore Bioassays

To measure LF performance, freshly hatched LF neonates were allowed to feed on WT and ko-*pik2l* rice plants. The larval mass (to an accuracy of 0.1 mg) was individually measured 11 days later. Each treatment was replicated 48~51 times.

### 4.10. JA, JA-Ile, and IAA Analysis

For JA, JA-Ile, and IAA analysis, 30-day-old plants of the WT and ko-*pik2l* rice lines were randomly assigned to LF and control treatments as described above. The youngest fully expanded leaf of each plant was harvested at different time points (0, 1, 3, and 8 h after the start of treatment) and was ground in liquid nitrogen. JA, JA-Ile, and IAA were extracted from a sample of about 100 mg of ground rice with ethyl acetate containing labeled internal standards (D^6^-JA, D^6^-JA-Ile, and D^5^-IAA) and then quantified by high-performance liquid chromatography–mass spectrometry–mass spectrometry (HPLC-MS-MS) (Agilent Technologies, Santa Clara, CA, USA) using the method described in [60]. Each treatment was replicated 4~6 times.

### 4.11. Analysis of the Activity of TrypPIs

To analyze TrypPIs, 30-day-old plants of the WT and ko-*pik2l* rice lines were randomly assigned to LF and control treatments as described above. The youngest fully expanded leaf of each plant was harvested at 0 and 2 d after the start of treatment and was ground in liquid nitrogen. Samples containing about 100 mg of ground rice were homogenized with 300 μL of cooled extraction buffer (0.1 M Tris-HC1, pH 7.6, 5% polyvinylpolypyrrolidone (Sigma-Aldrich, Darmstadt, Germany), 2 mg/mL phenylthiourea (Sigma-Aldrich, Darmstadt, Germany), 5 mg/mL diethyldithiocarbamate (Sigma-Aldrich, Darmstadt, Germany), and 0.05 M Na_2_EDTA) as described previously [61]. TrypPI activity was measured by the radial diffusion method [62]. Each treatment was replicated 7 times.

### 4.12. Detecting Flavonoids

To detect flavonoids, 30-day-old plants of the WT and ko-*pik2l* rice lines were randomly assigned to LF and control treatments as described above. The youngest fully expanded leaf of each plant was harvested at 0 and 2 d after the start of treatment and was ground in liquid nitrogen. Flavonoids were extracted from samples of approximately 100 mg using 800 μL of 70% methanol, and then quantified by HPLC-MS-MS as previously described in [63]. The content of each compound was calculated using an external standard method [64]. Each treatment was replicated 5~6 times.

### 4.13. Data Analysis

Two-treatment data were analyzed using Student’s *t*-tests (equal SDs) or *t* tests with Welch’s correction (unequal SDs). For comparisons between data collected from WT plants and from two transgenic lines of *OsPik-2-like* (ko-*pik2l*), an a priori approach based on Bayesian analysis of variance was used. All statistical analyses were conducted with SPSS software version 26 (IBM Corp., Armonk, NY, USA).

## Data Availability

The data presented in this study are available on request from the corresponding author.

## Supplementary Materials

The following supporting information can be downloaded at: https://www.mdpi.com/article/10.3390/plants13233275/s1, Figure S1: Sequences of nucleotides and deduced amino acids of OsPik-2-like; Figure S2: Sanger sequencing results of potential off-target sites; Figure S3: Other compounds in the leaves of WT plants and ko-*pik2l* lines; Table S1: Student’s *t*-test or *t*-test with Welch’s correction analysis with data from Figure 3; Table S2: Bayesian analysis of variance with data from Figure 4; Table S3: Bayesian analysis of variance with data from Figure 5; Table S4: Student’s *t*-test analysis with data from Figure S3; Table S5: Bayesian analysis of variance with data from Figure 6 and Figure S3; Table S6: Bayesian analysis of variance with data from Figure 7; Table S7: Primers used for real-time qPCR; Table S8: Primers used for *OsPike-2-like* cloning and subcellular localization assay; Table S9: Primers used for generation and characterization of transgenic plants.
