# Supplementary material for: A Nucleotide-Binding Domain Leucine-Rich Repeat Gene Regulates Plant Growth and Defense Against Chewing Herbivores
Source: Plants (Basel). 2024 Nov 22;13(23):3275. doi: 10.3390/plants13233275 (PMC11644264; doi:10.3390/plants13233275)
Supplement: Supplementary file 1 [file plants-13-03275-s001.zip › plants-3297467-supplementary.pdf]

plants

Supplementary data

A Nucleotide-Binding Domain Leucine-Rich Repeat Gene Regulates Plant Growth and Defense Against chewing herbivores

Chen Qiu [1], Xiaochen Jin [1], Yumiao Zhao [1,2], Peng Kuai[1]* and Yonggen Lou [1,2]*

1 State Key Laboratory of Rice Biology & Ministry of Agriculture Key Laboratory of Agricultural Entomology, Key Laboratory of Biology of Crop Pathogens and Insects of Zhejiang Province, Institute of Insect Sciences, Zhejiang University, Hangzhou 310058, China; cqiu2019@163.com (C.Q.); 12116098@zju.edu.cn (X.J.); zhaoyumiaoi@163.com (Y.Z.)
2 Hainan Institute, Zhejiang University, Sanya 572025, China
* Correspondence: kpchen7493@163.com (P.K.); yglou@zju.edu.cn (Y.L.); Tel.: +86-137-3551-5845 (P.K.); +86-571-88982622 (Y.L.)

Figure S1. Sequences of nucleotides and deduced amino acids of OsPik-2-like.

Figure S2. Sanger sequencing results of potential off-target sites.

Figure S3. Other compounds in the leaves of WT plants and ko-*pik2l* lines.

Table S1. Student's *t*-test or *t* test with Welch's correction analysis with data from Figure 3.

Table S2. Bayesian analysis of variance with data from Figure 4.

Table S3. Bayesian analysis of variance with data from Figure 5.

Table S4. Student's *t*-test analysis with data from Figure S3.

Table S5. Bayesian analysis of variance with data from Figure 6 and Figure S3.

Table S6. Bayesian analysis of variance with data from Figure 7.

Table S7. Primers used for real time-qPCR.

Table S8. Primers used for *OsPike-2-like* cloning and subcellular localization assay.

Table S9. Primers used for generation and characterization of transgenic plants.

```
1     ATGGAGGCAACGGCGGTGAGCTTGGCGCGGTCGGTGCTGGACGGCGTGCTGAACAGGGCGGGCTCCGCCGTCGCCGACGAGGCGGCGCTG
1      M  E  A  T  A  V  S  L  A  R  S  V  L  D  G  V  L  N  R  A  G  S  A  V  A  D  E  A  A  L

91    CTGCTTGGCGTGCGGCGGGAGGTGGAGTTCATCCGCGACGAGCTGGACATGATGAGATCATTCCTCAAGGTGGCCACGGCGAACCCCGAC
31     L  L  G  V  R  R  E  V  E  F  I  R  D  E  L  D  M  M  R  S  F  L  K  V  A  T  A  N  P  D

181   GCCGACGACACCGTGCGGACGTGGGTGAAGCAGGTGCGCGACCTCGCCTACGACGTCGAGGACTCCCTCCTCGACTTCGCGCTCTTCGCC
61     A  D  D  T  V  R  T  W  V  K  Q  V  R  D  L  A  Y  D  V  E  D  S  L  L  D  F  A  L  F  A

271   GACACGTTGTCGTCGTCCTCCTCGTCGTCGTGGTGGCTCCCGTGGCGCATCGCCGAGCGCCACCGCGTCGCGGCGAGGATCCGGGAGCTG
91     D  T  L  S  S  S  S  S  S  S  W  W  L  P  W  R  I  A  E  R  H  R  V  A  A  R  I  R  E  L

361   AAGGCGAGCGTCGAGGAGCTCAACCAGCGCTTCCTTCGGTACCGCATCGTCGTCGAACACCCGCGCGCGTCGCGCGGCGGCGGCGCCTCC
121    K  A  S  V  E  E  L  N  Q  R  F  L  R  Y  R  I  V  V  E  H  P  R  A  S  R  G  G  G  A  S

451   GACGACCAGCAGCAGCTTCACGACCACGATGGGCAGTACTACTCGGCGGAGCTGGCGTTCCAGGAGTCGGACATCATCGGGCGGGCGAGG
151    D  D  Q  Q  Q  L  H  D  H  D  G  Q  Y  Y  S  A  E  L  A  F  Q  E  S  D  I  I  G  R  A  R

541   GAGAAGGCGGAGGTGACGGCGCTCGTCCTCTCCGGCTGCGGCGGCGGCGACGTCGTCGGCGGCGGCGCGCTGGGGGTGGTGTCGGTGTGG
181    E  K  A  E  V  T  A  L  V  L  S  G  C  G  G  G  D  V  V  G  G  G  A  L  G  V  V  S  V  W

631   GGGATGGGCGGGATGGGGAAGTCGTCGCTGGTGCGGATGGTGTACAACGACCCGGAGCTCCTCGACGCCTTCGACTGCGACGCGTGGGTG
211    G  M  G  G  M  G  K  S  S  L  V  R  M  V  Y  N  D  P  E  L  L  D  A  F  D  C  D  A  W  V

721   ACGGTGCCGCACCCGCTCGACAGCGCCGACGAGTTCGTGCGGCGGCTGCGGCGCCACCTCGCCGTCGGCAAGGACCAGGACGTGCACGCC
241    T  V  P  H  P  L  D  S  A  D  E  F  V  R  R  L  R  R  H  L  A  V  G  K  D  Q  D  V  H  A

811   TACCTCCGGGAGAAGCGGTACGTGATCATCGTCGACGACCTGCACAGCCGGGAGGAGTGGGAGCACATCTGGCCGGTGCTGCACGTCGAC
271    Y  L  R  E  K  R  Y  V  I  I  V  D  D  L  H  S  R  E  E  W  E  H  I  W  P  V  L  H  V  D

901   GGCGGCAAGGGCAGCCGCGTGGTGGTGACGACGCGGCGGGAGGACGTCGCGCGCCACTGCGCGGGGCTCGTGCGCGAGGGGCACGGCCAT
301    G  G  K  G  S  R  V  V  V  T  T  R  R  E  D  V  A  R  H  C  A  G  L  V  R  E  G  H  G  H

991   GTCTACGAGCTCAGGCCGCTTGGGCGGGAAGAGTCAAAGGATCTCTTTTGTCAGAAGGTCTACAAATCAACCGAGTACATATTGGAGAAG
331    V  Y  E  L  R  P  L  G  R  E  E  S  K  D  L  F  C  Q  K  V  Y  K  S  T  E  Y  I  L  E  K

1081  GAAATGGAGGATCTAGCTGGTCCTATCTTAAAGAGATGCCGGGGGCTTCCACTGGCCATATCCACTATAGGAGGTCTGTTAGCCAATAGA
361    E  M  E  D  L  A  G  P  I  L  K  R  C  R  G  L  P  L  A  I  S  T  I  G  G  L  L  A  N  R

1171  CCCAAAACAGGCATAGAGTGGATAAAGTTGGATGAACACCTTGGTGCAGAGCTGGAGTCGTCTGACCTTCGTAACATCACTAAAGTGATT
391    P  K  T  G  I  E  W  I  K  L  D  E  H  L  G  A  E  L  E  S  S  D  L  R  N  I  T  K  V  I

1261  GTTTCAAGTTATGATGGCTTGCCATATTATTTGAAATCAATATTCTTATACCTCAGTATTTTCCCCGAGAATCATGAGATCAGATGCACC
421    V  S  S  Y  D  G  L  P  Y  Y  L  K  S  I  F  L  Y  L  S  I  F  P  E  N  H  E  I  R  C  T

1351  CGCTTGTTGAGGCGTTGGATGGCAGAAGGGTTCATAGCAAAGAACCGTGATATGCCGGTGGAGGAAGTGGGGCAGCGTTTCTACAATGAG
451    R  L  L  R  R  W  M  A  E  G  F  I  A  K  N  R  D  M  P  V  E  E  V  G  Q  R  F  Y  N  E

1441  CTCATCAACAGAAGTATGATCCAACCCTCCAAGAAGAGGATTAGCCCCAGCGTGAGTGTGGATCGCTGTCGGGTTCACAGCATGGTGCTA
481    L  I  N  R  S  M  I  Q  P  S  K  K  R  I  S  P  S  V  S  V  D  R  C  R  V  H  S  M  V  L

1531  CAGATCATCCTGTCCAAGTCCATTGAGGAGAACCAACTCTTCATCATCAAGAAACACTGCAATGAGGTTCCACAAAGTAAGATACGCCAT
511    Q  I  I  L  S  K  S  I  E  E  N  Q  L  F  I  I  K  K  H  C  N  E  V  P  Q  S  K  I  R  H

1621  CTGGTAGTATCCAGGTGGAAAAGGAGGGATGAGAGGCTAGAAAATATAAATTTTTCGTATGTTCGATCACTAACAGTGTTTGGGGATTGC
541    L  V  V  S  R  W  K  R  R  D  E  R  L  E  N  I  N  F  S  Y  V  R  S  L  T  V  F  G  D  C

1711  CCTGCTTCTCTCATATCTCCTAAAATGCGTCTTCTGCGAGTTCTGGACCTGGAAGATTCACTTAACCTGAAGAATGAAGACCTCAGGCAC
571    P  A  S  L  I  S  P  K  M  R  L  L  R  V  L  D  L  E  D  S  L  N  L  K  N  E  D  L  R  H

1801  GTAGGAGAGCTTCACCATCTAAGGTATCTTTGTTTGAGAGGTACAGAGATATCAAAGCTTCCATCTTTTCAGAACCTTCGGTACCTCGAG
601    V  G  E  L  H  H  L  R  Y  L  C  L  R  G  T  E  I  S  K  L  P  S  F  Q  N  L  R  Y  L  E

1891  ACGCTAGACATCCAAGACACAAAGGTGACACAGCTCCCTGATGGAATTGCAAAACTTGAGAAGCTACGCTATCTTCTTGCTGGCGTCAAC
631    T  L  D  I  Q  D  T  K  V  T  Q  L  P  D  G  I  A  K  L  E  K  L  R  Y  L  L  A  G  V  N

1981  TTCTCAAAAGAGTTGCTGCACAAGGTGGAACAGCCGGAGACAGATAATCGCAAGGCCAACCAGTTGGGCAACATGTTATCATGCTTATAT
661    F  S  K  E  L  L  H  K  V  E  Q  P  E  T  D  N  R  K  A  N  Q  L  G  N  M  L  S  C  L  Y

2071  TGCAACAGCAGTGATTATTGCGGGATTTCTAGCTTGGATCGAGTTAGTGTAAGAGCTCCTGAGGGAGTTGAGAAGCTAAGGGATTTGCAC
691    C  N  S  S  D  Y  C  G  I  S  S  L  D  R  V  S  V  R  A  P  E  G  V  E  K  L  R  D  L  H

2161  ATGTTGGGTGTGATCAATGTTGGCCATGGCAATGGAGTGGTAGGGAAGATTAAGAAGCTGACAAACTTGCGAAGGTTAGGAGTCTCAGGT
721    M  L  G  V  I  N  V  G  H  G  N  G  V  V  G  K  I  K  K  L  T  N  L  R  R  L  G  V  S  G

2251  GTCCTTAAGGAAGAAGGGCAGGACTTATGCAAGTCAATAGAGAAGCTAAGTCGTTTGCAGCGGCTTGAGCTGCGCTCTGATTCCCTAAAG
751    V  L  K  E  E  G  Q  D  L  C  K  S  I  E  K  L  S  R  L  Q  R  L  E  L  R  S  D  S  L  K

2341  TTTCTTGCTGAAAGTGAATTCGCAGCACCAAAACATCTTCTTTCACTTAGGCTATATGGCAATCTAGTCAGGTTGCCTAAATGGATTGGT
781    F  L  A  E  S  E  F  A  A  P  K  H  L  L  S  L  R  L  Y  G  N  L  V  R  L  P  K  W  I  G

2431  TCACTCAATGATCTGGCGAAGCTGAAGCTGCTGGGGACACAATTGAAGCAGGGAGAAATCATGCACCTCGGGAAGCTGCGCAACCTAGCA
811    S  L  N  D  L  A  K  L  K  L  L  G  T  Q  L  K  Q  G  E  I  M  H  L  G  K  L  R  N  L  A

2521  TTTCTCGGCTTGTGGGACAACTCTTATGTAGGGTATTCACTGCATTTTGGTCCTGGCACATTCCCAAAACTCAAGTTCCTTGACATTGAT
841    F  L  G  L  W  D  N  S  Y  V  G  Y  S  L  H  F  G  P  G  T  F  P  K  L  K  F  L  D  I  D

2611  GGGCTGAAGAACATTGAAACGGTGGCGATCGAGAATGGCGCGATGCCTGAGCTTGAACAACTCTGGGTGAACGACTGCAAAGGACTACTT
871    G  L  K  N  I  E  T  V  A  I  E  N  G  A  M  P  E  L  E  Q  L  W  V  N  D  C  K  G  L  L

2701  GATAGCAAAGATGGTTTATCAGGAGTACCACATCTGACAAACCTGAATGAGCTTCTTGTGAAGAAGTGTGGTGAGAAGGAGAACCTGATG
901    D  S  K  D  G  L  S  G  V  P  H  L  T  N  L  N  E  L  L  V  K  K  C  G  E  K  E  N  L  M

2791  GAGATACTGCAAACACAGGTCAGCGAACACAGTAAACGCCCGAAGTTCCTCATTGGTAAATCCATTGTAACAACAAGCCCCAAACGACAG
931    E  I  L  Q  T  Q  V  S  E  H  S  K  R  P  K  F  L  I  G  K  S  I  V  T  T  S  P  K  R  Q

2881  CGAACAGAGTAA
961    R  T  E  *
```

Figure S1. Sequences of nucleotides and deduced amino acids of OsPik-2-like.

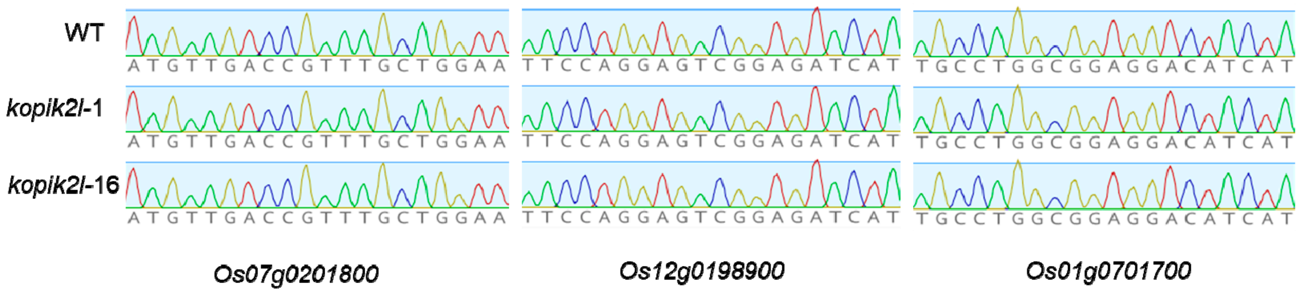

Figure S2. Sanger sequencing results of potential off-target sites.

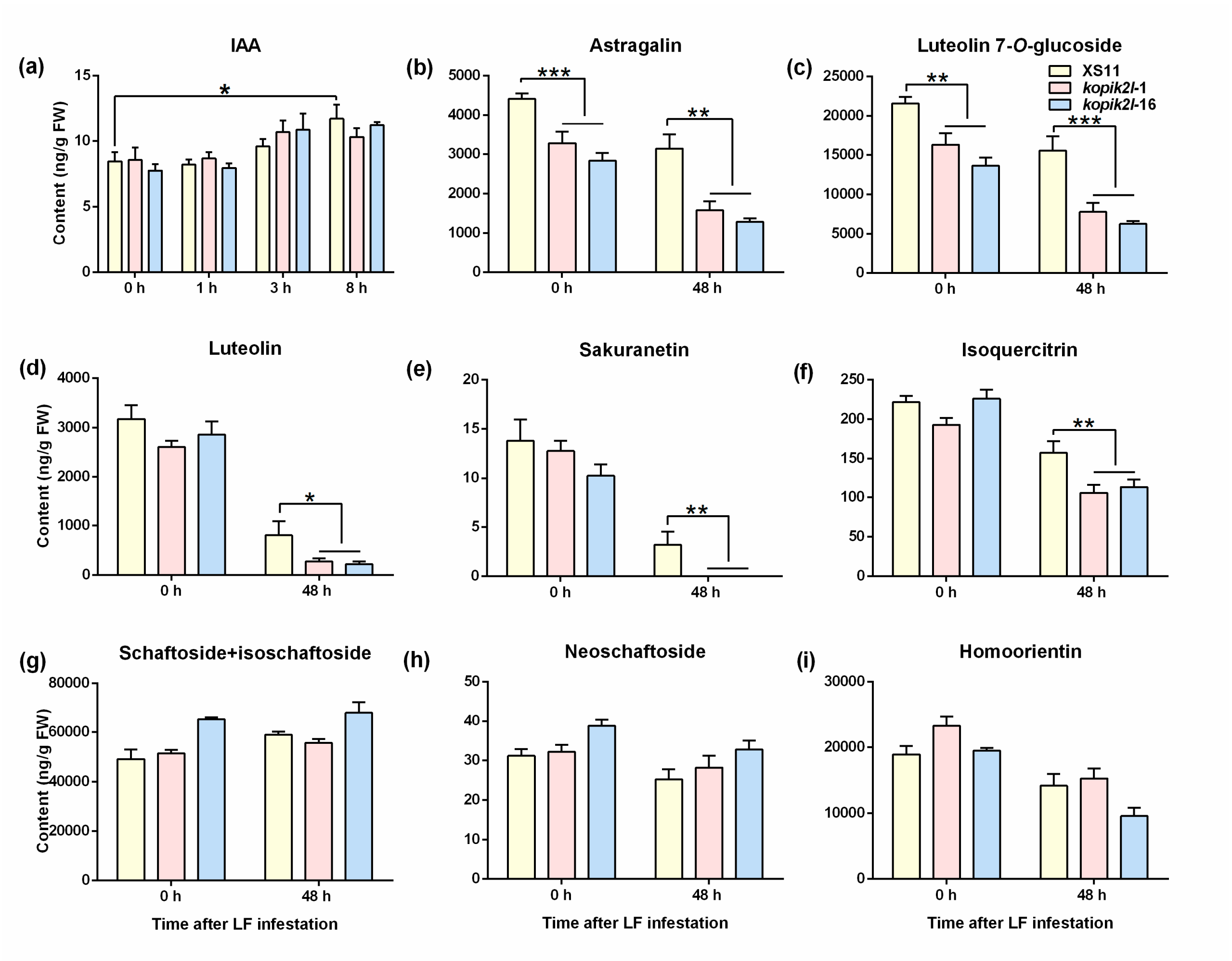

Figure S3. Other compounds in the leaves of WT plants and ko-*pik2l* lines. Mean levels (+SE, n = 4~6) of IAA (a), astragalin (b), luteolin 7-*O*-glucoside (c), luteolin (d), sakuranetin (e), isoquercitrin (f), schaftoside+isoschaftoside (g), neoschaftoside (h) and homoorientin (i) in WT plants and ko-*pik2l* lines that were individually infested by a 3rd instar LF larva on the first fully expanded leaf at the indicated time points. Asterisks indicate significant differences in ko-*pik2l* lines compared with WT plants evaluated by Bayesian analysis of variance or Student's *t*-tests (* $P < 0.05$, ** $P < 0.01$ and *** $P < 0.001$).

Table S1. Student's *t*-test or *t* test with Welch's correction analysis with data from Figure 3

Figure		*t*/ *Welch-corrected t*		*df*	*P* value
Figure 3b	0.5 h	*t*	4.098	7	0.0046
	1 h	*Welch-corrected t*	3.310	4.269	0.0269
	3 h	*Welch-corrected t*	1.439	4.014	0.2234
	8 h	*Welch-corrected t*	2.908	2.027	0.0991
	24 h	*Welch-corrected t*	3.031	4.006	0.0387
	72 h	*Welch-corrected t*	5.128	4.014	0.0068
Figure 3c	0 h	*t*	0.0	10	> 0.9999
	0.5 h	*Welch-corrected t*	28.79	4.444	<0.0001
	1 h	*Welch-corrected t*	5.293	5.464	0.0025
	3 h	*t*	1.688	9	0.1256
	8 h	*t*	1.772	7	0.1197
	12 h	*t*	1.084	7	0.3145
	24 h	*t*	2.013	7	0.0840
	48 h	*t*	8	8	0.3387
Figure 3d	0 h	*t*	0.0	9	> 0.9999
	0.5 h	*t*	1.900	8	0.0940
	1 h	*t*	1.909	8	0.0927
	3 h	*t*	0.3676	8	0.7227
	8 h	*t*	7	7	0.0908
	12 h	*Welch-corrected t*	4.253	5.679	0.0061
	24 h	*t*	1.538	8	0.1627
	48 h	*t*	0.4597	8	0.6580

Table S2. Bayesian analysis of variance with data from Figure 4

Figure	Parameter (XS11 vs. ko-*pik2l*)	*df*	*F*	*P* value	Bayes Fator-JZS
Figure 4a	Shoot height	1	14.428	<0.001	57.935
Figure 4b	Root length	1	31.880	<0.001	27572.426
Figure 4c	Chlorophy ll content	1	26.034	<0.001	4040.985
Figure 4d	Stem strength	1	5.031	0.029	1.093
Figure 4e	Shoot fresh weight	1	89.436	<0.001	3.749E+10
Figure 4f	Root fresh weight	1	62.123	<0.001	108537998.2
Figure 4g	Shoot dry weight	1	64.234	<0.001	178429062.1
Figure 4h	Root dry weight	1	75.531	<0.001	2221725275

Table S3. Bayesian analysis of variance with data from Figure 5

Compounds (XS11 vs. ko-*pik2l*)		*df*	*F*	*P* value	Bayes Fator-JZS
JA	0 h	\	\	\	\
	1 h	1	1.763	0.207	0.431
	3 h	1	0.793	0.388	0.274
	8 h	1	5.031	0.029	1.093
JA-Ile	0 h	1	0.062	0.807	0.207
	1 h	1	0.245	0.629	0.219
	3 h	1	1.044	0.324	0.307
	8 h	1	7.301	0.022	2.930
IAA	0 h	1	0.169	0.688	0.217
	1 h	1	0.055	0.818	0.200
	3 h	1	0.944	0.348	0.293
	8 h	1	1.190	0.301	0.369

Table S4. Student's t-test analysis with data from Figure S3

Compounds			*t*	*df*	*P* value
IAA	XS11	0h vs. 1h	0.3206	8	0.7567
		0h vs. 3h	1.295	8	0.2313
		0h vs. 8h	2.694	7	0.0309

Table S5. Bayesian analysis of variance with data from Figure 6 and Figure S3

Compounds (XS11 vs. ko-*pik2l*)		*df*	*F*	*P* value	Bayes Fator-JZS
Prunin	0 h	1	25.614	<0.001	162.070
	48 h	1	3.488	0.081	0.834
Carlinoside	0 h	1	10.410	0.007	7.543
	48 h	1	3.122	0.098	0.722
Isovitexin	0 h	1	7.348	0.018	3.173
	48 h	1	0.005	0.943	0.184
Schaftoside+isoschaftoside	0 h	1	4.481	0.054	1.237
	48 h	1	0.659	0.430	0.251
Neoschaftoside	0 h	1	2.782	0.119	0.654
	48 h	1	2.905	0.109	0.661
Homoorientin	0 h	1	2.350	0.149	0.550
	48 h	1	0.825	0.378	0.271
Astragalin	0 h	1	23.797	<0.001	120.790
	48 h	1	12.253	0.004	12.031
Luteolin 7-*O*-glucoside	0 h	1	15.018	0.002	20.682
	48 h	1	31.151	<0.001	366.566
Luteolin	0 h	1	2.149	0.168	0.519
	48 h	1	7.003	0.018	2.916
Sakuranetin	0 h	1	1.477	0.248	0.392
	48 h	1	11.193	0.004	10.169
Isoquercitrin	0 h	1	0.802	0.387	0.283
	48 h	1	10.759	0.005	9.023
TrypPI	0 h	/	/	/	/
	48 h	1	7.827	0.010	4.032

Table S6. Bayesian analysis of variance with data from Figure 7

Parameter	*df*	*F*	*P* value	Bayes Fator-JZS
larval mass	1	9.796	0.002	7.225

Table S7. Primers used for real time-qPCR

Gene names	ID	Forward primer (5′-…3′)	Reverse primer (5′-…3′)
OsActin	LOC_Os03g50885	TGGACAGGTTATCACCATTGGT	CCGCAGCTTCCATTCCTATG
OsPik-2-like	*Os09g0517200*	TGAAACGGTGGCGATCGAGAATG	TGCAGTCGTTCACCCAGAGTTG

Table S8. Primers used for *OsPike-2-like* cloning and subcellular localization assay

Names	Forward primer (5′-…3′)	Reverse primer (5′-…3′)
M13	GTAAAACGACGGCCAGT	CAGGAAACAGCTATGAC
OsPike-2-like-CDS	TCTCCGCCATTTACACCCAC	TGCCAAAGAGGAACATCGGA
1301-GFP sequencing	GTTCCCACTGAATCAAAGGC	CATGTTTGACAGCTTATCATCG
OsPike-2-like-GFP	aagcttatcgataccgtcgac ATGGAGGCAACGGCGGTG	atggtggcgaccggtaccgc CTCTGTTCGCTGTCGTTTGGG

Table S9. Primers used for generation and characterization of transgenic plants

Names	ID	Forward primer (5′-…3′)	Reverse primer (5′-…3′)
OsPike-2-like-OsU6b	*Os09g0517200*	gttgTTCCAGGAGTCGGACATCAT	aaacATGATGTCCGACTCCTGGAA
Cas9 test	/	TTCGACCAGTCCAAGAACGG	CTTGACCTTGGTGAGCTCGT
Hpt test	/	ACACTACATGGCGTGATTTCAT	TCCACTATCGGCGAGTACTTCT
OsPike-2-like test	*Os09g0517200*	CATTCCTCAAGGTGGCCACG	AGGCTTCCCTTGTATCCCCC
Off-target test 1	*Os07g0201800*	GAGAACATCACTCACCCGCG	CCACTCCTATCCCCCTTGCA
Off-target test 2	*Os12g0198900*	CTTGAGGACGTGGGTGAAGC	AACTGGAACGGAGGAGGGAG
Off-target test 3	*Os01g0701700*	GCATTGCGTGATCACAGGGA	AGGGGGCTCGTGCTTGATAT
